# Patterns of acute poisoning with pesticides in the paediatric age group

**DOI:** 10.1186/s12245-017-0148-5

**Published:** 2017-07-11

**Authors:** Kavinda Chandimal Dayasiri, Shaluka F. Jayamanne, Chamilka Y. Jayasinghe

**Affiliations:** 1 University Paediatrics Unit, Lady Ridgeway Hospital for Children, Colombo, Sri Lanka; 2 Faculty of Medicine, University of Kelaniya, Kelaniya, Sri Lanka

**Keywords:** Pesticide poisoning, Patterns, Children

## Abstract

**Background:**

Pesticides are identified as one of the dangerous poisons globally in children and are associated with increased short- and long-term morbidity. Pesticide poisoning is the most common method of self-poisoning among adults in rural Sri Lanka, and the clinical management is associated with significant healthcare costs to the country. There is however little data published on acute pesticide poisoning among children in rural Sri Lanka. The current study aimed to comprehensively evaluate clinical profiles, harmful first aid measures, emergency clinical management, complications and outcomes related to acute pesticide poisoning among children in the rural community of Sri Lanka.

**Methods:**

This multicenter study was conducted in the North Central Province of Sri Lanka involving all children with acute pesticide poisoning and who were between 9 months and 12 years of age. Data were collected over 7 years (2007–2014), and children from 36 hospitals were recruited. Data collection was carried out by pretested, multi-structured, interviewer-administered questionnaires to identify clinical profiles of children, harmful first aid measures, emergency clinical management, reasons for delayed management, complications and outcomes of pesticide poisoning events.

**Results:**

Among 1621 children with acute poisoning, 9.5% (155) comprised children with acute pesticide poisoning. Male children outnumbered female children, and the majority of children were less than 5 years. Most common pesticides implicated in poisoning of children were organophosphates and carbamates. Gastrointestinal and neurological symptoms were predominant clinical features. Limited transport and lack of concern regarding urgency among caregivers were leading reasons for delayed management. Most common location for poisoning was cultivation lands. Harmful first aid measures were practiced in 32.4%. 7.1% had intentional pesticide poisoning. The case fatality rate of all pesticide poisonings in the study was 1.9%. 58.1% of patients were transferred between regional hospitals and teaching hospital. Cardiac and respiratory arrests, aspiration pneumonia and convulsions were among the reported complications.

**Conclusions:**

Acute pesticide poisoning in paediatric age group (<12 years) is a relatively uncommon yet significant cause of child health-related morbidity and mortality in rural Sri Lanka. Patterns of poisoning represent the pattern of pesticide use by the rural community. The practice of harmful first aid measures by caregivers and delay in attending the emergency department may negatively impact patient outcomes.

## Background

Acute poisoning is one of the most common medical emergencies in young children and accounts for one of the leading causes for emergency department visits among adolescents [1]. Globally, more than one million children die following injuries every year [2], and poisoning is identified as the fourth leading cause of injury-related mortality in children [3]. The global mortality rate following acute poisoning in children younger than 20 years is 1.8 per 100,000 population [4]. Mortality rate is fourfold higher in low-middle income countries compared with that in developed countries [3, 4].

Pesticides are identified as one of the dangerous poisons in the paediatric age group [5]. Insecticides are among the common poisoning substances in children less than 5 years in South Asia [6]. While intentional poisoning of pesticides is associated with high mortality [7], unintentional poisoning which is usually seen among children carries low mortality. Recent studies indicate that children can be exposed to pesticides during oral and manual exploration in the surrounding environment [8]. Young children tend to have increased access to pesticides among rural populations where the caretakers belong to farming community.

Pesticide drift refers to unintentional diffusion of pesticides in environment and is a recognized mode of accidental pesticide poisoning. Children can be poisoned with pesticides passively via pesticide drift, through breastfeeding by farm worker mothers and contamination. Organophosphates and carbamates account for the highest number of pesticide-related poisoning events in children [9]. Acute toxicity with these pesticides is associated with long-term neuropsychiatric health-related adverse effects in the affected person [10].

Pesticide poisoning is the most common mode of intentional poisoning among Sri Lankan adults [11]. Most households store pesticides within or immediately outside the house, and lockable containers are infrequently used by rural communities [12]. Easy accessibility and popularity were the two key determinants in intentional pesticide poisoning among adults in rural community of Sri Lanka [11]. Expenditure for managing these self-poisoned patients was significant that an average of US$ 31.83 was spent per patient in addition to cost of transferring them for specialized care which was US$ 14.03 per patient [13]. Data regarding pesticide poisoning among children of rural Sri Lanka is limited. Pesticides were among the three commonest toxic agents used for unintentional poisoning in children in western province of Sri Lanka [14]. Another study from urban Sri Lanka reported in 2006 that agrochemicals accounted for 6% of poisoning events and that study did not observe fatalities related to pesticide poisoning [15]. Since pesticide poisoning is most frequent in rural community of Sri Lanka and patterns of poisoning are likely to change over time, the children of rural Sri Lanka are likely to show different poisoning patterns. There are not any studies from rural communities of Sri Lanka which have evaluated patterns and risk factors for pesticide poisoning among children. In this back ground, the current study aimed to identify clinical profiles, poison-related factors, harmful first aid measures, clinical management, complications, outcome and risk factors of acute pesticide poisoning among children in the rural community of Sri Lanka.

## Methods

### Study setting

This multicenter study was based in the North Central Province (NCP) of Sri Lanka which accommodates a predominantly rural population. The study included 36 hospitals in NCP (Anuradhapura Teaching Hospital (THA), Polonnaruwa District General Hospital (PDGH) and 34 base/district/rural hospitals of the province under RDHS (Regional Director of Health Services)).

### Study design

Clinical profiles, poison characteristics, circumstances of poisoning and trends related to acute pesticide poisoning among children were evaluated over a period of 7 years (2007–2014). The pesticides considered were herbicides, rodenticides, insecticides and fungicides. The study was conducted in four major arms—(1) a 2-year prospective study (2012–2014) at Anuradhapura Teaching Hospital, (2) a 2-year prospective study (2012–2014) at Polonnaruwa District General Hospital, (3) a 1-year prospective study involving 34 hospitals under RDHS of NCP (2013–2014) and (4) a 5-year retrospective study at Anuradhapura Teaching Hospital (2007–2012). All three prospective studies identified clinical manifestations of pesticide poisoning and the reasons for delayed management (defined for the study as a delay of more than 90 min to present at primary care hospital following the poisoning event) in addition to basic demographic data.

### Participants

The study recruited all children who were between 9 months and 12 years of age. Children with both intentional and unintentional pesticide poisoning were considered for data analysis. Acute poisoning due to non-pesticide poisons (household poisons/medicines/poisonous plants), food poisons, snake envenomation, allergic reactions and adverse drug reactions which can be considered in the purview of toxicology were omitted in the study. Also, children with doubtful poisoning where there was no clear aetiology were excluded from the study.

### Data collection

#### THA prospective series

Data were collected from the caregivers of children who met inclusion criteria. Mothers were interviewed in most encounters, and fathers or other caregivers were interviewed only when mothers were not available to participate in the study. Major part of the data collection was conducted at Anuradhapura Teaching Hospital, and data collection from all caregivers in the prospective study in that setting was done by the principal investigator himself to minimize interviewer bias. Interviews with the caregivers were conducted on the same day of admission to minimize possible recall bias. Data were collected using a pretested, multi-structured questionnaire (annex 1) which comprised of questions to identify demographic data, type and circumstances of poisoning, poison-related factors, location of poisoning, harmful first aid measures, clinical management, reasons for delayed management, complications and outcome following acute poisoning. The questionnaire was pretested by administration of the questionnaire to 20 caregivers in the same study setting over 4-month period prior to commencement of the study and expert review. Extensive local and international literature survey was done prior to drafting of the questionnaire. A total of 383 children presented with acute poisoning over a 2-year study period, and 346 children had poisoning with an agent other than a pesticide. Two children with doubtful pesticide were also excluded. Thirty-seven children, in whom the diagnosis of acute pesticide poisoning was confirmed, were recruited to the study.

#### PDGH and RDHS prospective series

Clinical research associates carried out data collection at PDGH and RDHS over 2 and 1 year duration respectively. Data collections were done prospectively as defined in the study protocol. All clinical research associates were trained by the principal investigator to administer questionnaires to minimize interviewer bias. Piloting was carried out in both study settings for 4 months prior to commencement of the study, and all data collections were done under direct supervision of the investigators of the study.

A total of 371 children presented with acute poisoning over the 2-year study period at PDGH. Three hundred twenty-five children had poisoning with an agent other than a pesticide and were excluded. Forty-six children with confirmed acute pesticide poisoning were recruited to the study. Similarly, 242 children presented with acute poisoning over 1 year at RDHS. Twenty-three children were recruited after exclusion criteria.

#### THA retrospective series

Retrospective study was conducted based on bed head ticket (BHT) data, and only limited demography- and poison factor-related data which could be considered reliable and auditable by discharge registers were collected. Data in the retrospective series were collected by the principal investigator himself to minimize record retrieval-related bias. Out of 625 children who had acute poisoning, 49 children with definite acute pesticide poisoning were recruited to the study over the 5-year study period.

### Data analysis

All data were analysed using SPSS version 19.0.

### Data reliability and auditing

Data collections in all components of the current study were subjected to independent audit and close monitoring by the South Asian Clinical Toxicology Research Collaboration (SACTRC) and the investigators of the study.

### Ethical approval

Ethical clearance for the study was issued by the ethical review committees, Faculty of Medicine, University of Kelaniya and Rajarata University of Sri Lanka. Written informed consent was obtained from participant children’s parents or guardians.

## Results

Over a period of the 7-year study period, 1621 children presented with acute poisoning. One hundred fifty-five children (9.5%) who had acute pesticide poisoning were recruited to the study. Male children outnumbered female children in all arms of the study and amounted to 92 (59.4%). The majority of children were below 5 years—114/155 (73.6%). Most poisoning events were secondary to unintentional ingestion of the pesticide (140/155, 91.3%). Prospective series at Anuradhapura Teaching Hospital identified six children (27%) with intentional pesticide ingestion which was comparatively a higher proportion compared to proportions observed in the other arms of the study. Mortality rate was 1.9% (3 cases), and all three children (100%) had ingested a lethal dose of organophosphate. Two children who succumbed had ingested the poison intentionally. Fifty-eight percent of children (90/155) were transferred from a local hospital (under RDHS) to a tertiary care hospital following the poisoning event. Majority of children belonged to the farming community and at least one parent was engaged in farming activities in 90/ 155 (58.1%) families. 77/155 families (49.7%) had been using pesticides for farming activities. Table 1 compares the demographic characteristics, patterns of pesticide poisoning and transfer rates of children in different arms of the study.

**Table 1:** Demographic characteristics, patterns of poisoning and transfer rates of children with pesticide poisoning

Variable	Retrospective study (*n* = 49)	THA study (*n* = 37)	DGHP study (*n* = 46)	RDHS study (*n* = 23)	Total (*n* = 155)
1. Male/female	25:24 51%:49%	23:14 62.1%:37.9%	27:19 58.7%:41.3%	17:6 73.9%:26.1%	92:63 59.4%:40.6%
2. <5 years:>5 years	40:9 81.6%:18.4%	25:12 65.6%:34.4%	31:15 67.3%:32.7%	18:5 78.2%:21.8%	114:41 73.6%:26.4%
3. Unintentional/intentional	47:2 95.9%:4.1%	31:6 83.2%:16.2%	44:2 95.6%:4.4%	22:1 95.6%:5.4%	144:11 92.9%:7.1%
4. Mortality	1 (2%)	1 (2.7%)	1 (2.17%)	–	3 (1.9%)
5. Most common poison	Carbamates	OP	OP	OP	OP
6. Transfer rate	28 (57.1%)	30 (81.1%)	17 (37.0%)	15 (65.2%)	90 (58.1%)

*DGHP* District General Hospital Polonnaruwa, *RDHS* Regional Director of Health Services, *THA* Anuradhapura Teaching Hospital, *OP* organophosphate pesticides

The most common pesticide implicated in poisoning of children were the organophosphates, and it accounted for 63/155 (40.6%) poisoning events. Prospective series at Anuradhapura Teaching Hospital however observed carbamates as the commonest pesticide implicated in 20 (40.7%) poisoning events. Overall, carbamate insecticide and herbicide poisonings were commonly observed after organophosphate poisoning. Poisoning with those three pesticides accounted for 76.2% of total pesticide poisonings. Three deaths were reported over the 7-year study period. Two children died following cholinergic crisis leading to respiratory failure. The other child died following severe aspiration pneumonia and chemical pneumonitis following excessive administration of water (>500 ml) to induce emesis, and the water was offered as a home remedy by caregivers. Table 2 presents all children with poisoning based on the pesticide ingested.

**Table 2:** Demographic characteristics and patterns of poisoning of children with pesticide poisoning

Pesticide	THA retrospective series (*n*—49)	THA prospective series (*n—*37)	DGHP prospective series (*n*—46)	RDHS prospective series (*n*—23)	Total (*n*—155)
1. Organophosphate insecticides	10 (20.4%)	17 (45.9%)	24 (52.1%)	12 (52.1%)	63 (40.6%)
2. Carbamate insecticides	20 (40.7%)	5 (13.5%)	4 (8.6%)	7 (30.4%)	36 (23.3%)
3. Herbicides	4 (8.2%)	5 (13.5%)	9 (19.5%)	1 (4.3%)	19 (12.3%)
4. Rodenticides	6 (12.3%)	3 (8.1%)	3 (24.1%)	1 (4.3%)	13 (8.4%)
5. Pyrethroid insecticides	4 (8.2%)	2 (5.4%)	4 (8.6%)	2 (8.6%)	12 (7.7%)
6. Fungicides	–	3 (8.1%)	–	–	3 (1.9%)
7. Unknown pesticides	5 (10.2%)	2 (5.4%)	2 (4.3%)	–	9 (5.8%)

*DGHP* District General Hospital Polonnaruwa, *RDHS* Regional Director of Health Services, *THA* Anuradhapura Teaching Hospital

Commonly reported organophosphate insecticides were Pyrinex chlorpyrifos and malathion. Herbicides included propanil, glyosphosphate and paraquat. ‘Roundup’, ‘MCPA’ and ‘Nominee’ were among the identified trade names. Commonly seen fungicides included ‘Captan’ (phthalimide fungicides). Ant chalk was the commonly seen pyrethroid insecticide. Kuretor insecticide, which is a crystal that is used to kill harmful subterranean pests that attack the roots of banana and destroy the plants, was associated with 19 poisonings (12.4% overall) of children.

Ingestion was the most common route of poisoning (152/155, 98.1%). Two children had symptoms following contact of the pesticide with the mucus membranes, and one child had inhaled the pesticide before becoming symptomatic.

### Comparison of clinical manifestations and reasons for delayed presentations to primary care hospital

One hundred six children recruited to prospective studies at THA (*n*—37), PDGH (*n*—46) and RDHS (*n*—23) were available for the analysis. The majority of children remained asymptomatic after pesticide poisoning. Gastrointestinal symptoms (39 children, 36.8%) were the predominant symptoms following acute pesticide ingestion, and it was consistently seen in all three studies. Most gastrointestinal symptoms occurred following organophosphate and carbamate intoxication. Among those who developed symptoms, 72.7% had gastrointestinal symptoms and 37.3% developed neurological symptoms following ingestion of agrochemical poisons. Table 3 illustrates the variability in clinical manifestations in detail.

**Table 3:** Clinical manifestations of pesticide poisoning among children in rural Sri Lanka

Clinical manifestations	THA	PDGH	RDHS	Total
1. Gastrointestinal symptoms	12 (32.4%)	19 (41.3%)	8 (34.7%)	39 (36.8%)
2. Neurological symptoms	7 (18.9%)	8 (17.3%)	5 (21.7%)	20 (18.9%)
3. Respiratory symptoms	4 (10.8%)	4 (8.6%)	2 (8.7%)	10 (9.4%)
4. Cardiovascular symptoms	2 (5.4%)	2 (4.2%)	–	4 (3.8%)

Fifty-six children (52.8%) presented to primary care hospital at least 1 h after the ingestion of the poison, and 20 children (18.9%) were brought at least 2 h after the poisoning event. Most common reason for delayed management was delayed presentation to primary care hospital due to lack of concern regarding urgency of the situation—24 children (22.6%). Detailed analysis of reasons for delayed management is presented in Table 4.

**Table 4:** Reasons for delayed management of children with acute pesticide poisoning in rural Sri Lanka

Reasons for delayed presentation	THA	THP	RDHS	Total
1. Lack of concern regarding urgency of the situation	9 (24.3%)	8 (17.3%)	7 (30.4%)	24 (22.6%)
2. Lack of transport facilities in emergencies	8 (21.6%)	8 (17.3%)	5 (21.7%)	21 (19.8%)
3. Lack of knowledge regarding possible complications	6 (16.2%)	6 (13%)	7 (30.4%)	19 (17.9%)
4. Lack of financial resources	6 (16.2%)	5 (10.8%)	3 (13%)	14 (13.2%)
5. Child had not told about the incident until symptoms occur	2 (5.4%)	2 (4.3%)	1 (4.3%)	5 (4.7%)
6. Delayed attention by the medical team	1 (2.7%)	–	–	1 (0.9%)

### THA prospective series

Over the 2-year study period, 37 children presented to THA following acute pesticide poisoning. The mean age of children was 4.9 years (range 13 months–12 years). Most parents had received secondary education—27 fathers (73%) and 28 mothers (75.6%). The majority of fathers were engaged in farming (18, 48.6%). Most mothers were housewives (21, 56.8%); however, ten mothers (27%) were regularly engaged in farming activities. Most of the poisoning events occurred in the cultivation area (19, 51.3%) followed by home garden (7, 18.9%) and home kitchen (6, 16.2%) where the pesticides had been stored unsafely.

Harmful first aid measures were practiced in 12 children (32.4%). The most common measure was forceful ingestion of plain water (5, 13.5%), and it was followed by forceful soap water ingestion (3, 8.1%) and forceful coconut milk ingestion (2, 5.4%). In 89% (11/12) of these children, the caregivers were unaware of the detrimental effects of these potentially harmful first aid practices. Emesis induction was offered to 20 children (54%). Five children (13.5%) required prescription of atropine to antagonize the toxic effects of organophosphate pesticides. Four children (10.8%) needed management in an intensive care unit. Reported medical complications included cardiac arrhythmia (2, 5.4%), aspiration pneumonia (2, 5.4%), seizures (2, 5.4%) and respiratory arrest (1, 2.7%).

## Discussion

Pesticides are widely used in both agricultural and domestic settings globally. Both active and passive exposures however are associated with adverse health-related outcomes in children. Widespread and largely unregulated use of pesticides by rural communities of Sri Lanka has increased the burden of acute pesticide poisoning [16] and emerged as one of the major public health problems [17]. Most of these observations were made by adult studies in Sri Lanka, and to date, there are only few studies conducted on children in rural communities where the adult population is found to be at high risk for pesticide poisoning.

The current study found that pesticide poisoning among children was relatively uncommon (9.4% of total poisoning events). The majority of children were below 5 years. These observations have been similarly reported in other studies [18]. Organophosphates and carbamates were implicated in 63.9% (99/155) of acute pesticide poisoning events and were the most common poisoning agents. It is consistently seen in similar studies [19].Over the 7-year study period, organophosphates emerged as the most common poison as compared with carbamates which was the commonest poison during the initial years. This observation may represent the trends in pesticide use over time by rural communities with increasing use of organophosphates compared to carbamates.

Few studies from developed countries have seen ocular contamination and dermal exposure as common routes of poison exposure compared to ingestion [20]. However, current study found that most children ingested the poison and it had been the most frequent route of poisoning. This observation is similarly made by other studies [21].

A prospective study of adults in the same population in Sri Lanka evaluated by the current study reported that approximately 50% of patients were transferred from regional hospitals to the teaching hospital for further management [22]. The average transfer rate of paediatric patients in the current study was 58.1%. The difference could be attributed to relatively limited training to health care workers in regional hospitals in managing paediatric patients with acute pesticide poisoning. The case fatality rate was 1.9%, and it was less than the case fatality rates among adults with acute pesticide poisoning [23]. Deliberate self-poisoning by children below 12 years is little documented in wider international literature. Krishnakumar et al. reported nine children with intentional pesticide poisoning in their case series in 2005 [24]. The current study reported 11 children with intentional pesticide poisoning.

Many patients in the current study were administered home remedies. These may result in harm or may result in delay to care. The evaluation of circumstances of poisoning and data on mortality revealed that harmful remedies including forceful administration of liquids lead to severe aspiration pneumonia and death. Therefore, provision of health education to at-risk communities regarding these harmful home remedies may reduce childhood poisoning-related morbidity and mortality.

Variation in the manifestations of symptoms following acute pesticide poisoning depends on the type and quantity of the poison ingested. As poisoning patterns vary over geographic areas, the symptom profiles of paediatric poisoning studies also show great variation. Non-specific gastrointestinal manifestations were the most common symptoms following pesticide poisoning. Similar clinical manifestations have been observed in adults following acute pesticide exposure [25]. Neurological manifestations were observed after ingestion of organophosphate pesticides, and it is consistent with other paediatric studies in literature [26].

Children in the current study were exposed mostly to agriculture-related pesticides, and the majority of events occurred in cultivation areas. Similar studies [20] from different geographic regions have observed household insecticides as more common poisons than agriculture-related pesticides and most events occurred in the household. These differences represent the wide variation in patterns of pesticide poisoning in children.

Analysis of reasons for delayed presentation to primary care unit revealed that lack of caregivers’ concern regarding urgency to seek medical care and lack of transport facilities in emergencies are the major barriers for timely management. In most peripheral locations, the public transport systems are not available for rural dwellers during nighttime and people hardly move out owing to the fear of wild animals. Need for long-distance travelling had reportedly been an obstacle for managing adult patients with poisoning in rural Sri Lanka [27]. Financial resources are also a limiting factor, and the majority of people cannot owe for private transport. Scarcity of educational and communication resources limit accessibility to information [28]. Similar studies from South Asian subcontinent [29] reported that the delay in presentation to the hospital could be due to ignorance, poverty, insufficient knowledge and lack of easy modes of transportation. The mean duration for the presentation at the primary care hospital was more than 5 h in studies from other regions in South Asia [28, 30, 31]. The current study, however, noted that more than 80% children were brought to primary care unit within 2 h despite bearing the same limiting factors.

There is emerging evidence that pesticide exposure during childhood is associated with an increased risk of acute lymphocytic leukemia, brain tumors [18] and adverse neurodevelopmental outcomes [32]. Pesticide exposure can also be a trigger factor for precipitating wheezing exacerbations in children with bronchial asthma [33]. Poisoning event, moreover, is invariably a psychologically traumatizing event to both the child and his caregivers. This growing body of evidence highlights the fact that despite most children not having acute complications immediately following the poisoning event, they are at high risk for long-term complications. It is important that all child health care providers are well aware of these factors and every child with acute pesticide poisoning needs to be taken very seriously. All such children need to be directed for neurodevelopmental evaluation, and the whole family may need continuous support and guidance from the paediatrician over a long time.

## Conclusions

Acute pesticide poisoning in paediatric age group (<12 years) is a relatively uncommon yet significant cause of child health-related morbidity and mortality in rural Sri Lanka. Patterns of poisoning represent the pattern of pesticide use by the rural community (Table 5). The practice of harmful first aid measures by caregivers and delay in attending the emergency department may negatively impact patient outcomes.

**Table 5:** Pattern of location of poisoning, harmful first aid measures and complications of pesticide poisoning among children at THA

Characteristic of poisoning	Variable	No.	Percentage
1.Location of poisoning event	Cultivation area	19	51.3%
	Home garden	7	18.9%
	Home kitchen	6	16.2%
2.First aid measures	Forceful ingestion of plain water	5	13.5%
	Forceful ingestion of soap water	3	8.1%
	Forceful ingestion of coconut milk	2	5.4%
3.Complications	Cardiac arrhythmia	2	5.4%
	Aspiration pneumonia	2	5.4%
	Seizures	2	5.4%
	Respiratory arrest	1	2.7%
